# The Molecular and Cellular Basis of Regional Itch Differences

**DOI:** 10.21203/rs.3.rs-6466187/v1

**Published:** 2025-05-26

**Authors:** Santosh Mishra, Joshua Wheeler, Nidha Williams, Thierry Olivry

**Affiliations:** North Carolina State University; North Carolina State University; North Carolina State University; Department of Clinical Sciences, College of Veterinary Medicine, NC State University

## Abstract

Itch is a complex, noxious sensation associated with many skin and systemic conditions, which varies in intensity and quality across different body regions. Despite its prevalence, the molecular and cellular mechanisms underlying regional itch differences remain poorly understood. Here, we investigate the neural basis of regional itch differences, focusing on the role of neuropeptides and histamine receptors expression. Using a combination of behavioral, pharmacological, genetic, histological, and molecular approaches, we identified distinct regional differences in itch response between the cheek and dorsal nape (neck). Our findings reveal that Substance P modulates itch in the cheek, while histamine receptor 1 (HRH1) is the primary receptor mediating itch but not pain in the cheek. We also discovered regional differences in sensory neuron subpopulations, with increased co-expression of HRH1 and neuropeptides in trigeminal ganglion neurons. In brief, we provide new insights into the molecular and cellular mechanisms underlying regional itch differences, highlighting the importance of considering regional variations in itch response to develop effective treatments.

## INTRODUCTION

Somatosensory neurons in the dorsal root ganglia (DRG) and trigeminal ganglia (TG) detect various stimuli, generating distinct sensations and responses. DRG neurons innervate the skin of the limbs and torso, whereas TG neurons innervate unique sensory areas in the head and neck, including the eyes, nose, mouth, meninges, and vibrissae. Reports have identified genes linked to somatosensory neurons’ detection specificity in DRG and TG. DRG RNA-sequencing reveals distinct expression profiles for proprioceptive and itch-responsive neurons predominantly in mice ^1,2^ and humans^3–5^. However, TG neuronal populations differ from DRG, as they lack proprioceptive neurons and innervate several unique sensory environments: the eyes, nasal passages and sinuses, meninges, and, in animals, vibrissae. Recent research has found TG-specific differences at the transcription level^4,6,7^, but whether that induces a differential behavioral response or differences in itch circuitry in mice is unknown.

In humans, the severity of itch is perceived differentially across body regions, with the face and torso^8–10^ exhibiting distinct intensity thresholds. Animal models have recapitulated these phenomena, showing fewer scratching bouts in response to intradermal pruritogen injections in the cheek than dorsal nape^11,12^. This difference is consistent across common pruritogens^11–19^, including histamine^11,12^. Notably, facial injection of pruritogens and algogens activates an overlapping population of second-order neurons found in the spinal trigeminal nucleus^15^. In contrast, itch and pain are processed in the DRG sensory neurons and activate separate populations of second-order neurons^19,20^.

The distinct neural processing of itch in the TG versus the DRG suggests the involvement of specific neuropeptides and signaling pathway involved in transmitting itch signals from the face versus the body. Itch and pain sensations are mediated through specific neuropeptides. Substance P (SP) mediates pain sensations^21^. Meanwhile, itch sensations are mediated by somatostatin (SST)-expressing neurons, which are > 99% co-localized with brain natriuretic peptide (BNP)-expressing neurons^20,22^. Activation of these first-order neurons triggers the release of these neuropeptides, which then activate second-order neurons in the dorsal horn of the spinal cord to further process these pain and itch sensations^19,20,22,23^.

The significant difference in itch processing between the face (cheek) and torso (neck) reveals a substantial knowledge gap. To address this, we employed a multifaceted approach combining mouse genetics and behavioral, pharmacological, cellular, and molecular techniques. Following intradermal histamine injections into the dorsal nape or cheek, we investigated itch behavior in C57Bl6/J (inbred) and CD-1 (outbred) mice. Additionally, we examined histamine-induced itch behavior in mice lacking BNP or SP as well as in mice treated with antagonists for their respective receptors (NPR1^24^ and TACR1). We also assessed nape and cheek skin innervation density by genetically marking somatostatin-expressing neurons with tdTomato. To identify differences in neuronal populations, we used tracing techniques followed by immunohistochemistry and *in-situ* hybridization to analyze histamine receptors H1 and H3, BNP/SST, and SP in human and mouse DRG and TG. In summary, these experiments aim to elucidate the mechanisms underlying the differences in itch processing between the face and body.

## RESULTS

### Regional scratching response between the face and neck

**Figure 1:**
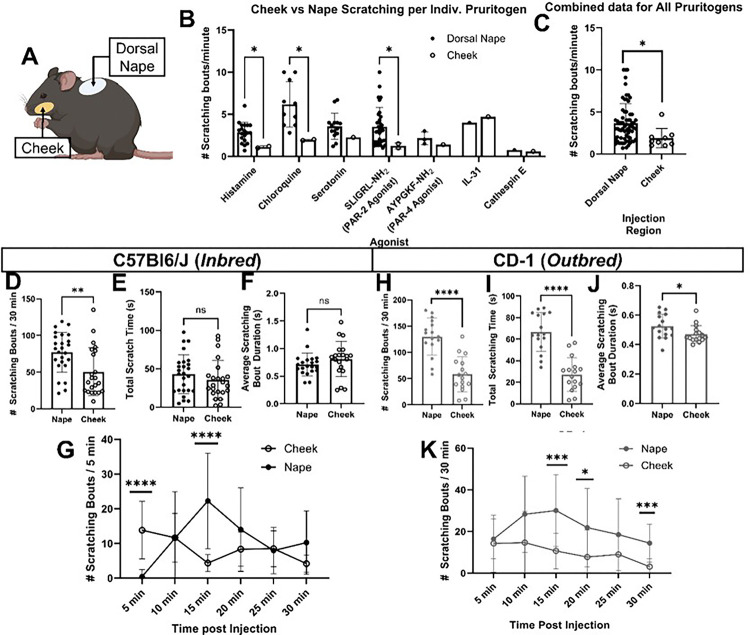
Scratching behavior differs significantly between the dorsal nape and cheek of inbred and outbred mice. **A)** Cartoon highlighting the dorsal nape and cheek injection sites. **B)** Results of a literature search for common pruritogens comparing the normalized itch behavior (scratching bouts/minute) of the dorsal nape and cheek reported in the literature. **C)** Compiled data from Panel A demonstrating that, regardless of pruritogen, the normalized amount of itch behavior reported is significantly reduced in the cheek as compared to the dorsal nape. **D)** Intradermal (i.d.) injection of histamine (100 μg/ 20 μL) induces significantly more scratching bouts in the dorsal nape (innervated by the DRG) than the cheek of C57Bl6/J mice. **E)**There are no significant differences in the total amount of time spent scratching the dorsal nape or cheek following i.d. histamine (100 μg/ 20 μL) in C57Bl6/J mice. **F)** There are no significant differences in scratching bout duration between the dorsal nape and cheek following i.d. histamine (100 μg/ 20 μL) in C57Bl6/J mice. **G)** Significantly more cheek-directed scratching bouts occur within the first 5 minutes following histamine (100 μg/ 20 μL) injection (i.d.), while significantly more dorsal nape-directed scratching bouts occur ~15 minutes following histamine (100 μg/ 20 μL) injection (i.d.). **H)** Intradermal (i.d.) injection of histamine (100 μg/ 20 μL) induces significantly more scratching bouts in the dorsal nape than the cheek of CD-1 mice. **I)** There is a significant differences in the total amount of time spent scratching the dorsal nape or cheek following i.d. histamine (100 μg/ 20 μL) in CD-1 mice. **J)** There are no significant differences in scratching bout duration between the dorsal nape and cheek following i.d. histamine (100 μg/ 20 μL) in CD-1 mice. **K)** Significantly more dorsal nape-directed scratching bouts occur 15 to 25 minutes following histamine (100 μg/ 20 μL) injection (i.d.) in CD-1 mice. Data is presented as Mean ± Standard Deviation. Each dot represents one biological replicate. Significance in Panels A & B were determined using a 2-tailed Mann-Whitney U-test. Significance for Panels C, D, E, G, H, & I were determined using a 2-tailed Student’s t-test. Significance in Panels F & J were determined using a 2-way ANOVA. ** p < 0.01, **** p < 0.0001.

We initially performed a literature review of papers published between January 2009 and June 2023 (Fig. 1B,C) which revealed significant differences in scratching bouts, when normalized for time, between these two regions in response to various pruritogens, including for histamine (*p < 0.0001), chloroquine (*p = 0.0008), and SLIGRL-NH2, PAR-2 specific agonists (*p = 0.0006) (Fig. 1B). This data was further confirmed when we combined all scratching bouts/minute values for all pruritogens (*p = 0.0073) (Fig. 1C).

To investigate the regional scratching response between the face and neck, we comprehensively analyzed scratching behavior in C57Bl6/J mice following intradermal injections of pruritogens into the dorsal nape and cheek. Histamine elicited significantly fewer scratch bouts of ipsilateral cheek-directed scratching than dorsal nape-directed scratching bouts (*p = 0.0040) (Fig. 1D) in C57Bl6/J mice injected with histamine. In contrast, we did not find any significant differences when comparing the total amount of time spent scratching directed at these areas (*p = 0.2947) (Fig. 1E), and no significant differences were seen in the average bout duration (*p = 0.2540) (Fig. 1F). The time course of histamine-induced scratching bouts differed significantly between the dorsal nape and cheek (Fig. 1G). Ipsilateral cheek-directed scratching bouts peaked within the first 5 minutes (*p < 0.0001) (Fig. 1G) and the number of dorsal nape-directed scratching bouts peaked at 15 minutes (*p = 0.0001) (Fig. 1G).

To confirm that our findings in C57Bl6/J mice were not strain-specific, we repeated the experiments using CD-1 mice, a commonly used itch research outbred mouse line (Fig. 1H–K)^26^. Our results showed a similar pattern: fewer ipsilateral cheek-directed scratching bouts (*p < 0.0001); however, we found significantly shorter scratching times than dorsal nape-directed scratching bouts (*p < 0.0001) (Fig. 1H &I). Unlike with C57BL/6J mice, bout duration significantly differed between regions in the CD-1 mice (*p = 0.0380) (Fig. 1J). The time course of histamine-induced scratching bouts differed between regions, with cheek-directed scratching peaking within 5 minutes, and significant differences were observed at 15 (*p = 0.0002), 20 (*p = 0.0477) and 30 minutes (*p = 0.0009) (Fig. 1K).

Overall, our data suggests that regional differences in itch are conserved across outbred and inbred mouse lines, with the cheek being less responsive to histamine-induced itch compared to the dorsal nape.

#### Regional itch differences are not explained by innervation densities

**Figure 2:**
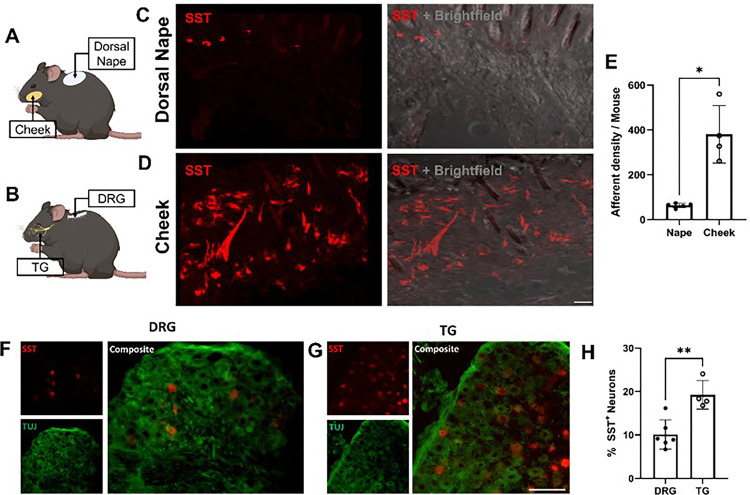
The cheek is more densely innervated by SST-positive neurons than the dorsal nape. **A)** Cartoon highlighting the dorsal nape and cheek locations. **B)** Cartoon highlighting the location of the TG and DRG. **C)**Representative confocal images of skin isolated from the dorsal nape of naïve SST-cre::Ai9 mice. **D)** Representative confocal images of skin isolated from the cheek of naïve SST-cre::Ai9 mice. **E)** Quantification of the innervation density of the dorsal nape and cheek of SST-cre::Ai9 mice. Data is presented as Mean ± Standard Deviation. F) Mouse schematic labeling the locations of the dorsal nape, cheek, DRG, and TG. G) Representative image of SST expression in the TG. H) Representative image of SST expression in the DRG. G) Quantification of SST-positive neurons in the DRG and TG, normalized to Tuj staining. Each dot represents one biological replicate. *p < 0.05 as determined by a paired 2-tailed Student’s t-test.

Given the difference in histamine-induced itch between the dorsal nape and cheek, we hypothesized that there may be a difference in innervation density of itch sensing neurons between the facial dermatomes innervated by the TG^27^ and the dorsal nape dermatomes innervated by the DRG^28^. To assess this, we generated SST-cre::Ai9 mice in which tdTomato was conditionally expressed in SST-expressing neurons. We chose to generate SST-cre::Ai9 mice since previous work has demonstrated that SST-expressing DRG sensory neurons are responsible for mediating itch signals induced by histamine^20,29^. We found that the skin of the cheek has a significantly higher degree of innervation by SST-cre::Ai9 neurons as compared to the dorsal nape (*p = 0.0158) (Fig. 2C - E). Due to the increased innervation density of SST-cre::Ai9 afferents in the cheek, we hypothesized that there was an increase in the number of SST-expressing neurons in the TG. As expected, we found significantly more SST-expressing neurons in the TG as compared to the DRG (*p = 0.0029) (Fig. 2F–H). This increase is likely to be responsible for increased SST-positive afferent density between the dorsal nape and the cheek. These findings also establish that the higher innervation density of itch-sensing neurons in the cheek does not correlate with the reduced scratching response observed in this region.

#### Distinct role of SP in modulating itch response in the cheek and dorsal nape.

Since innervation density did not explain the regional differences in itch response, we hypothesized that pain-associated neuropeptides, specifically Substance P (SP)^21^, might modulate ipsilateral cheek-directed scratching behavior. Based on previous observations where we found the dorsal nape, all SST-expressing DRG sensory neurons are positive for BNP^20^. Further, these BNP-expressing DRG sensory neurons are not positive for Substance P (SP)^22^, a canonical pain-associated neuropeptide.^21^ While there is literature about the relative co-expression of these neuropeptides in DRG sensory neurons, there is no information about their co-expression in TG sensory neurons.

**Figure 3:**
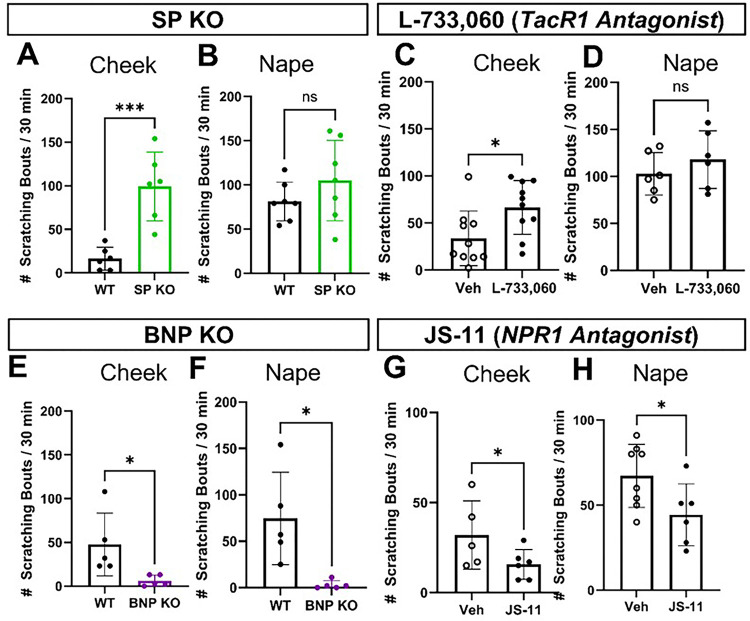
Loss of Substance P significantly increases the total number of ipsilateral-cheek directed scratching bouts. **A)** Substance P KO mice had significantly more ipsilateral cheek directed scratching bouts as compared to WT controls following intradermal histamine (100 μg/20 μL) injection. **B)**There were no significant differences in the total number of dorsal nape-directed scratching bouts between SP-KO mice and WT controls following intradermal histamine (100 μg/20 μL) injection. **C)** Pharmacological inhibition of TacR1 with L-733,060 (1 mg/kg in 100 μL I.V.) significantly increased the total number of ipsilateral cheek-directed scratching bouts following intradermal histamine (100 μg/20 μL) injection **D)**Pharmacological inhibition of TacR1 with L-733,060 (1 mg/kg in 100 μL I.V.) did not significantly change the number of dorsal nape-directed scratching bouts following intradermal histamine (100 μg/20 μL) injections. **E)** BNP-KO mice exhibited significantly fewer ipsilateral cheek-directed scratching bouts following intradermal histamine (100 μg/20 μL) injection. **F)** BNP-KO mice exhibited significantly fewer dorsal nape-directed scratching bouts following intradermal histamine (100 μg/20 μL) injection. **G)** Intraperitoneal administration of JS-11 (163 μg in 50 μL) did not significantly reduce the total number of ipsilateral cheek-directed scratching bouts following intradermal histamine (100 μg/20 μL) injection. **H)** Intraperitoneal administration of JS-11 (163 μg in 50 μL) significantly reduced the total number of dorsal nape-directed scratching bouts following intradermal histamine (100 μg/20 μL) injection. Data is presented as Mean ± Standard Deviation. *p < 0.05, **p < 0.01, ***p < 0.001 as determined by a 2-tailed Student’s t-test. Each data point represents 1 biological replicate.

To test if SP modulated itch responses in the cheek, we used knockout (KO) mice lacking SP or BNP and quantified scratching bouts after histamine injection into both the dorsal nape and the cheek (Fig. 3). We found that SP-KO mice exhibited significantly more ipsilateral cheek-directed scratching bouts than wild-type controls (*p = 0.0007) (Fig. 3A), with no differences in dorsal nape-directed scratching bouts (*p = 0.2390) (Fig. 3B). We used BNP-KO mice as a control, as these mice should lack an itch behavior phenotype, and demonstrated that the loss of BNP resulted in a significant decrease in the total number of dorsal nape (*p = 0.0126) *and* ipsilateral cheek-directed (*p = 0.0338) scratching bouts (Fig. 3E & F).

To confirm that our behavior results in SP-KO and BNP-KO mice, we pharmacologically inhibited the SP receptor (TacR1) with L-733,060^31,32^. We found that these mice exhibited significantly higher amounts of ipsilateral cheek-directed scratching bouts than vehicle-treated mice (*p = 0.0198) (Fig. 3C). Like the SP-KO mice, L-733,060 treated mice had no significant differences in dorsal nape-directed scratching bout compared to vehicle-treated mice (*p = 0.3568) (Fig. 3D). Mice treated with the NPR1 antagonist JS-11^33^ experienced significantly fewer scratching bouts in both the ipsilateral cheek (*p = 0.0338) and the dorsal nape (*p = 0.0396) (Fig. 3G & H). These findings suggest that SP plays distinct roles in modulating itch response in the cheek and dorsal nape, with SP inhibiting and BNP promoting itch in the cheek.

#### Histamine receptor-specific itch induction.

To investigate which histamine receptors, mediate histamine-induced itch in these regions, we built on our previous findings on regional differences in itch between the cheek and nape. We focused on the four histamine receptors, three of which are expressed in DRG sensory neurons. scRNA-sequencing data showed that HRH1 mRNA is expressed predominantly in SST- and MrgprA3-positive DRG sensory neurons, while HRH3 is expressed in SP-positive DRG sensory neurons involved in pain^2^.

**Figure 4:**
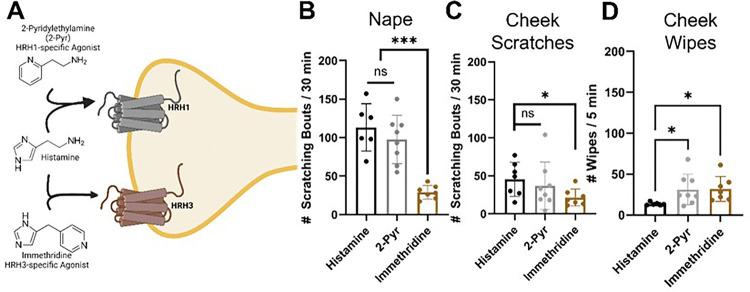
Specific activation of HRH3 with immethridine induced significantly fewer scratching bouts and significantly higher wiping bouts. **A)**Schematic demonstrating which histamine receptors are activated by the agonists Histamine, 2-pyridylethylamine, or immethridine. **B)** immethridine (100 μg/ 20 μL) induces significantly fewer scratching bouts than histamine (100 μg/ 20 μL) or 2-Pyridylethylamine in the dorsal nape. **C)** immethridine (100 μg/ 20 μL) induces significantly fewer scratching bouts than histamine (100 μg/ 20 μL) or 2-Pyridylethylamine in the ipsilateral cheek. **D)** immethridine (100 μg/ 20 μL) induces significantly more ipsilateral cheek directed wiping bouts than histamine (100 μg/ 20 μL). Data is presented as Mean ± Standard Deviation, *p < 0.05, ***p < 0.001 as determined by a 1-way ANOVA with a *posthoc* Holm – Šídák test for multiple comparisons. Schematic in panel **A** was made in BioRender.

To determine which histamine receptor(s) are responsible for transducing histamine-induced itch, we injected 2-pyridylethylamine (HRH1-specific agonist)^34^ and immethridine (HRH3-specific agonist)^35^ into the dorsal nape and cheek of mice. We found that 2-pyridylethylamine induced HRH1-mediated itch in both the dorsal nape and ipsilateral cheek, with no significant differences in scratching bouts compared to histamine-injected mice (*p = 0.5208, nape; *p = 0.7708, cheek) (Fig. 4B & C). We found that 2-Pyr induced significantly more cheek wipes as compared to histamine in addition to the cheek directed scratching (*p = 0.0230) (Fig. 4D). Immethridine also induced more ipsilateral cheek wipes (*p = 0.136) (Fig. 4D), but fewer scratching bouts than histamine (*p < 0.0001, nape; *p = 0.0264, cheek) in both regions. Immethridine induced fewer scratching bouts than 2-pyridylethylamine in the nape (*p = 0.0002, nape), but not the cheek (*p = 0.2373). Our results suggest that HRH1 is the primary histamine receptor mediating itch in dorsal nape-innervating DRG sensory neurons, while HRH3 may play a role in pain-behavior.

### Regional variations in sensory neuron populations underlying itch and pain

**Figure 5:**
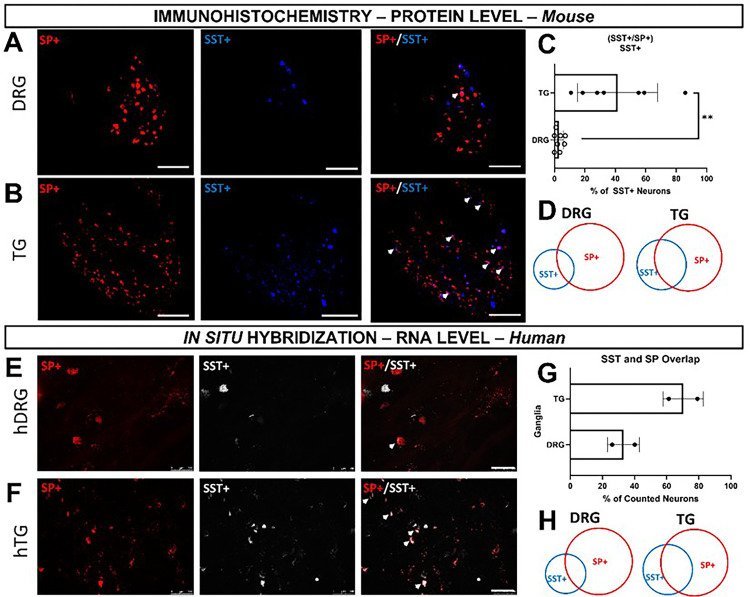
Protein and gene expression showing SST and SP overlap in the TG of mice and human. **A)** Representative immunohistochemical images of SST and SP positive DRG neurons isolated from Tac1-cre::Ai9 mice that were stained for SST. **B)** Representative immunohistochemical images of SST and SP positive TG neurons isolated from Tac1-cre::Ai9 mice that were stained for SST. **C)** Quantification of neurons positive both SST & SP normalized to the total number of SST-positive neurons. **D)** Venn Diagram demonstrates the relative percentage of DRG neurons positive for SST-only, SP-only, & both SST and SP. **E)** Venn Diagram demonstrates the relative percentage of TG neurons positive for SST-only, SP-only, & both SST and SP. **K)** Representative ISH images of SST and SP positive human-derived DRG neurons. **L)**Representative ISH images of SST and SP positive human TG neurons. **M)**Quantification of human DRG and TG neurons positive for SST, SP, or SST and SP normalized to the number of SST-positive neurons. **N)** Venn diagram demonstrating the relative percentage of human DRG neurons positive or SST, SP, or Both. **O)** Venn diagram demonstrates the relative percentage of human TG neurons positive for SST, SP, or both. Data is shown as Mean ± Standard Deviation, **p < 0.01 as determined by a 2-tailed paired Student’s t-test. Arrows identify neurons positive for both SST and SP. Scale bar = 100 μm for all images. Similar results were seen in N = 5 (mice) or N = 2 (human) samples.

Our previous findings on regional differences in itch and SST/SP innervation led us to investigate the populations of SST-, SP-, HRH1-, and HRH3-expressing sensory neurons in the dorsal root ganglion (DRG) and trigeminal ganglion (TG) of mice, which we also further validated in humans. Our SST-cre::Ai9 innervation results indicated that there should be a larger proportion of SST-positive TG sensory neurons. Additionally, our behavior results indirectly indicate that SP-positive TG sensory neurons are important in modulating ipsilateral cheek itch. Since pain signaling is capable of modulating itch sensation and vice versa^19^, we hypothesized that there is a change in the number of TG sensory neurons that are positive for both SST and SP. We found that overall, there was a significant increase in the percentage of TG sensory neurons positive for both SST and SP as compared to the DRG (*p = 0.0012) (Fig. 5A – D). We further validated this in human using *in situ* hybridization (ISH; Fig. 5E–H).

**Figure 6:**
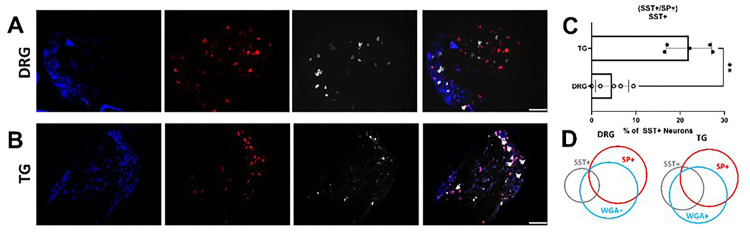
Retrograde tracing shows neurons that express both SST and SP in the TG of mice that innervate the skin. **A)** Representative *in situ* hybridization (ISH) images of SST and SP positive DRG neurons isolated from Wheat Germ Agglutinin injected (1mg/mL in 50 μL) C57Bl6/J mice that were stained for SST and SP. **B)** Representative immunohistochemical images of SST and SP positive TG neurons isolated from Wheat Germ Agglutinin injected (1mg/mL in 50 μL) C57Bl6/J mice that were stained for SST and SP. **C)**Quantification of neurons positive both SST & SP normalized to the total number of SST-positive neurons. **D)** Venn Diagram demonstrating the relative percentage of DRG neurons positive for WGA-only, SST-only, SP-only, & WGA, SST, and SP. **E)** Venn Diagram demonstrating the relative percentage of TG neurons positive for WGA-only, SST-only, SP-only, & WGA, SST, and SP. Data is shown as Mean ± Standard Deviation, **p < 0.01 as determined by a 2-tailed paired Student’s t-test. Arrows identify neurons positive for both SST and SP. Scale bar = 50 μm for all images. Similar results were seen in at N = 5 (mice) samples.

**Figure 7:**
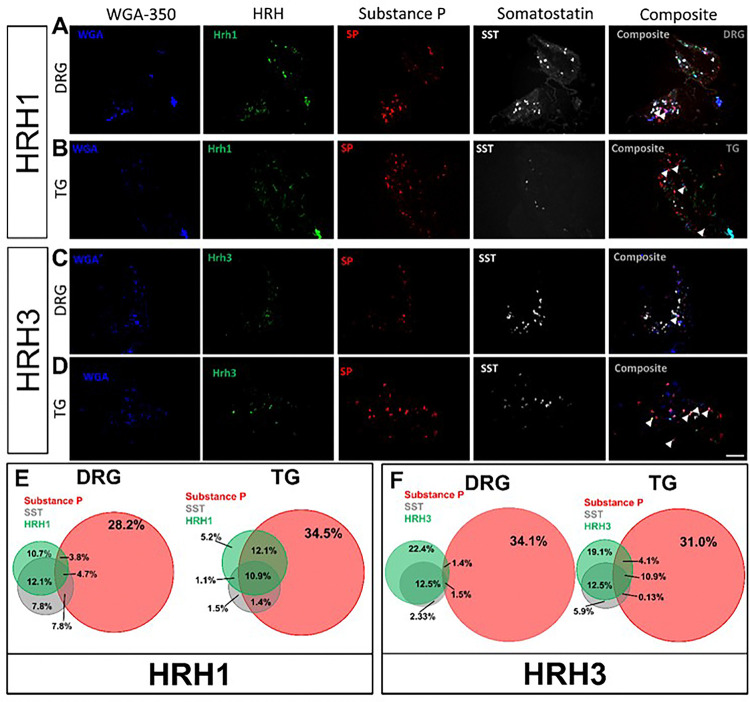
Skin-innervating TG sensory neurons have larger populations of neurons positive for HRH1, SST, and SP or HRH3, SST, and SP as compared to skin-innervating DRG sensory neurons. **A)** Representative images of DRG neurons isolated from mice receiving intradermal WGA-350 (50 μL of 1 mg/mL stock) that were ISH stained for HRH1, SP, and SST. **B)** Representative images of TG neurons isolated from mice receiving intradermal WGA-350 (50 μL of 1 mg/mL stock) that were ISH stained for HRH1, SP, and SST. **C)** Representative images of DRG neurons from mice receiving intradermal WGA-350 (50 μL of 1 mg/mL stock) that were ISH stained for HRH3, SP, and SST. **D)** Representative images of TG neurons from mice receiving intradermal WGA-350 (50 μL of 1 mg/mL stock) that were ISH stained for HRH3, SP, and SST. **E)** The Venn diagram showing the sizes of the HRH1, SP, and SST populations normalized to WGA-350-positive DRG neurons. **F)** The Venn diagram showing the sizes of the HRH1, SP, and SST populations normalized to WGA-350-positive TG neurons. **G)** The Venn diagram showing the sizes of the HRH3, SP, and SST populations normalized to WGA-350-positive DRG neurons. **H)** The Venn diagram shows the sizes of the HRH1, SP, and SST populations normalized to WGA-350-positive TG neurons. Scale bar = 100 μm. Percentages shown are the average of 5 male mice. Similar ISH staining patterns were seen in all 5 C57Bl6/J mice. Arrows identify neurons positive for all three genes of interest plus WGA-350.

**Figure 8:**
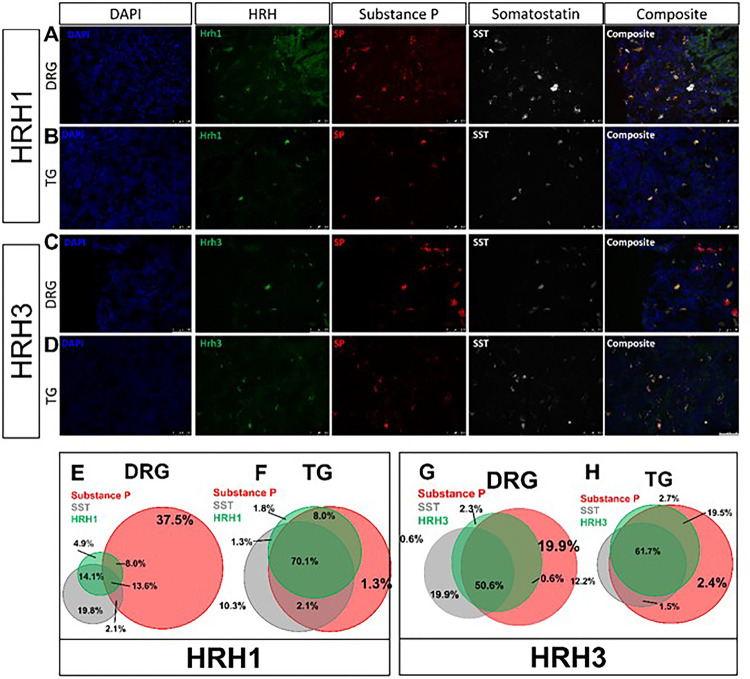
Human DRG and TG recapitulate the changes in DRG and TG sensory neuron populations of HRH1 or HRH3, SST, and SP. **A)**Representative FISH images of Human DRG neurons stained for HRH1, SP, and SST. **B)** Representative FISH images of Human TG neurons stained for HRH1, SP, and SST. **C)**Representative FISH images of Human DRG neurons stained for HRH3, SP, and SST. **D)** Representative FISH images of Human DRG neurons stained for HRH3, SP, and SST. **E)**Venn diagram showing the relative sizes of the HRH1, SP, and SST populations normalized to the number of counted DRG neurons. **F)** Venn diagram showing the relative sizes of the HRH1, SP, and SST populations normalized to the total number of counted TG neurons. **G)** Venn diagram showing the relative sizes of the HRH3, SP, and SST populations normalized to the total number of counted DRG neurons. **H)** The Venn diagram showing the sizes of the HRH3, SP, and SST populations normalized to the total number of counted TG neurons. Scale bar = 100 μm. Percentages shown are the average of 2 male human DRG or TG. Similar ISH staining patterns were seen in both DRG or TG human samples. Arrows identify neurons positive for all three genes of interest.

Using wheat germ agglutinin (WGA-350) as a neuronal tracer, used here to identify skin-innervating sensory neurons, we also found a larger population of cheek skin-innervating TG sensory neurons positive for both SST and SP compared to dorsal nape-innervating DRG sensory neurons (*p = 0.0051) (Fig. 6). Next, we investigated the expression of HRH1 and HRH3 in skin-innervating sensory neurons in mice (Fig. 7). Our results showed an increase in the percentage of TG sensory neurons expressing HRH1, SST, and SP, as well as HRH3, SST, and SP, compared to the DRG. We further corroborated these findings in human TG versus DRG regional-specific differences in human sensory neurons (Fig. 8).

## DISCUSSION

**Figure 9:**
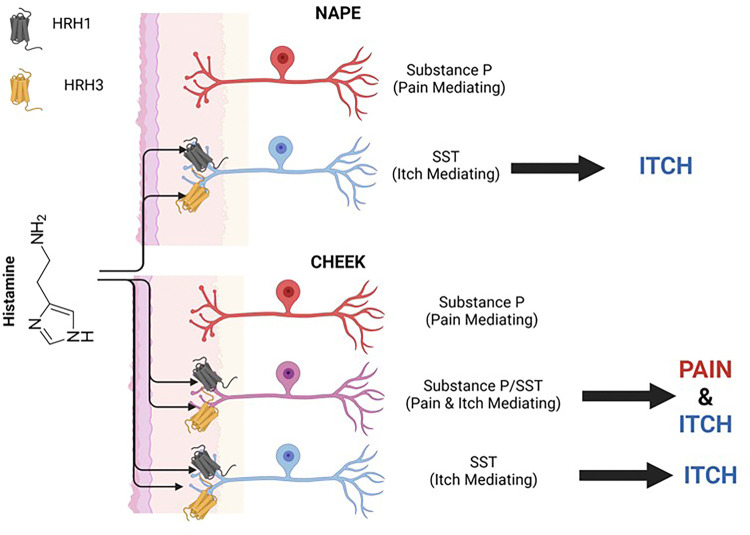
Schematic of histamine-evoked itch in the dorsal nape and cheek. Interstitial histamine activates HRH1 and/or HRH3 on DRG and TG sensory neurons. The majority of HRH1 and HRH3 expressions are limited to the SST population of itch mediating neurons in the DRG. In the TG, HRH1 and HRH3 are expressed on the SST-positive itch TG neurons and expressed in the SST-positive/SP-positive population of TG neurons that are polymodal for itch and pain signaling. The activation of these SST-positive/SP-positive neurons result in modulation of TG-mediated itch signals. Schematic was made in BioRender.

Our study reveals significant regional differences in itch response between the cheek and dorsal nape (Fig. 1). These differences are not attributed to variations in innervation density (Fig. 2), but to distinct populations of sensory neurons expressing specific neuropeptides and receptors (Fig. 3–4). Here, we demonstrate that the difference in facial histaminergic itch processing is due to differences in the proportion of TG neurons that co-express Substance P with SST, HRH1, and HRH3 (Figs. 5, 6 & 7)). Behaviorally, this results in the likely activation of HRH1, inducing pain responses in the cheek. Further, we demonstrate that HRH3 might transduce histamine activation into pain (Fig. 4). Our histological results were also recapitulated in human DRG and TG samples (Figs. 5 & 8), extending their translational relevance. Finally, our results indicate that the TG processes facial itches fundamentally differently by a mechanism similar to what is seen in the gate control model of itch and pain processing (Fig. 9).

Here, we confirmed that the number of histamine-induced ipsilateral cheek-directed scratching bouts is significantly reduced as compared to the number of histamine-induced dorsal nape scratching bouts by systematically comparing the scratching bouts by using the C57Bl/6J mice and the same amount of histamine in the same injection volume for both the cheek and nape injections. These results confirm that the reduction in the total number of ipsilateral cheek-directed scratching bouts is due to a difference in the neurophysiology of the cheek skin as opposed to differences in injection volumes or dose (at least for histamine). Interestingly, outbred CD-1 mice, which are thought to be a better model for human research^36^, had significantly fewer ipsilateral cheek-directed scratching bouts, corroborating the results from the inbred line. Furthermore, CD-1 mice have significantly shorter scratching bout durations (Fig. 1I), implying that they also perceive histaminergic itch in the cheek as less intense than histaminergic itch in the dorsal nape. Validation of regional differences in histamine-induced itch between inbred and outbred lines suggests that the study findings are not limited to a specific mouse strain and likely translatable to humans.

One of the limitations of our study is that we specifically investigated the role of histamine in regional itch differences, despite literature suggesting that other itch mediators, such as chloroquine, serotonin, SLIGRL-NH2, IL31, and proteases, may also exhibit similar regional differences (Fig. 1). There are mostly three reasons for this focus: Firstly, histamine is a well-known endogenous mediator for itch. Further, its involvement in itch processing is a helpful model for understanding the neural mechanisms underlying regional differences itch sensitivity. Secondly, in sensory neurons, histamine’s-induced itch primarily mediated by the histamine receptor 1 (HRH1). This receptor specificity allows for targeted investigation of histamine’s role in regional itch differences, without the confounding effects of other itch mediators. Lastly, histamine allows for a more in-depth investigation of the neural mechanisms underlying regional itch differences, which can serve as a foundation for future studies on other itch mediators, and the clinical relevance of histamine in itch disorders makes it a prime target for therapeutic intervention at sensory neurons carrying message to the CNS.

### Histamine-induced behavior hints at TG-specific processing of itch behavior

Reports have indicated differences between the DRG and TG at the transcriptome level^3,4,6^. Yet, the overall assumption has been that itch and pain processing in the TG follows the same pathways as itch processing in the DRG. Electrophysiology work performed by Akiyama et al., ^15^ further hinted at a difference in pain and itch signaling when they reported that pruritogens and algogens, itch inducers and pain inducers, respectively, results in postsynaptic activation of an overlapping population of neurons in the spinal trigeminal (TG) nucleus.

There are currently two main theories about how the nervous system processes itch and pain. The first is a modified label-line theory, which posits that itch and pain are processed by two parallel pathways by the peripheral nervous system and the spinal cord dependent on the input simulus^37,38^. Overall, the label line theory is correct for the DRG; SST-positive/BNP-positive neurons primarily process itch, and pain is mediated mainly by SP-expressing DRG neurons^21,22^. Further supporting this theory is the finding that activation of the MrgprA3-expressing DRG neurons by capsaicin in TRPV1-KO::MrgprA3^ROSA – TRPV1^ mice, wherein TRPV1 is *only in MrgprA3-expressing neurons*, results in itch – *not* pain^18^. The second theory of itch and pain processing is the Gate Control theory, which states that separate pain and itch-responding DRG neurons activate the same set of secondary neurons located in the dorsal horn of the spinal cord. This theory was founded by discovery of dynorphin-expressing spinal interneurons activated following TRPV1 or TRPM8 neurons activation^19^. Activation of these dynorphin-expressing interneurons triggers the release of dynorphin, which then suppresses activation of gastrin-related peptide receptor (GRPR)-expressing spinal interneurons, thus preventing the transmission of itch signals^19^. Current research has determined that these GRPR-expressing spinal interneurons are where itch signals converge^19,20,22,23,39,40^ before these interneurons transmit itch signals to the parabrachial nucleus^41,42^. This theory is further supported by the finding that SST-dependent activation of SSTR2-subpopulation inhibits pain responses ^20^. Both of these theories on itch processing have been assumed to be the *de facto* pathways by which the TG processes itch behavior despite the lack of evidence, either for or against, the presence of these pathways in the TG nucleus.

Our results suggest that *the TG itself uses gate control to process facial itch signals* despite the unclear presence of these pathways in the spinal trigeminal nucleus^43^. Our mouse data demonstrate that TG have a significantly increased percentage of SST-expressing neurons expressing Substance P compared to the DRG (Fig. 5). HRH1, which transduces histamine receptor activation into itch, has significant changes in downstream neuropeptide populations (Figs. 7 & 8). In the DRG, HRH1 is predominantly co-expressed with SST, with few neurons being positive for HRH1 and Substance P; however, in the TG, this proportion of HRH1-positive and Substance P-positive neurons roughly triples (Fig. 7). This change in HRH1 and Substance P expression also results in a change in behavioral outcomes (Fig. 4). HRH3 underwent similar changes in the proportion of neurons positive for Substance P in the TG compared to the DRG (Figs. 7 & 8). Interestingly, immethridine, an HRH3-specific agonist^35^, did not induce itch but significantly more ipsilateral cheek-directed wipes than histamine, indicating that histaminergic itch is both receptor-dependent and neuropeptide-dependent. These findings provide a basis for examining how the gate control theory of pain and itch processing may be relevant in this context. The gate control theory of pain and itch processing has evidence that there might be two subtypes: the leaky gate subtype suggesting itch and weak pain signals activate the same pathway^44^, and an intensity coding subtype suggests itch signal transmission is dependent on a frequency or burst of stimulation^45^. While we could not test this hypothesis here, but our results, and those reported in Akiyama et al^15^, alluding to an intensity coding in the TG. Another avenue to explore would be to determine if the histamine-triggered SP-release can activate a previously identified monosynaptic facial pain circuit^46^ as these neurons express the receptor for SP^47^. Activation of this pathway by histamine would also likely account for the decrease in itch behavior and increase in pain following intradermal injection of histamine into the cheek.

In summary, we identified distinct sensory neuron subpopulations, highlighting the importance of considering regional differences in itch response when designing treatment strategies. Overall, our study has significant implications for the understanding and treatment of itch disorders by developing therapies targeting HRH1 and HRH3 receptors that may provide new treatment options for itch disorders.

## METHODS

### Chemicals

Histamine was ordered from Sigma Aldrich (Catalog # H7125). Wheat Germ Agglutinin conjugated to Alexa Fluor 350 was ordered from Invitrogen (Catalog # W11263). L-733,060 (Catalog # 1145), 2-Pyrdylethylamine (Catalog # 2478), and immethridine (Catalog # 2315) were ordered from Tocris. JS-11 (1-cyclohexyl-3-(cyclopropylmethyl)-N-((3-methylisoxazol-5-yl)methyl)-2-oxo-2,3-dihydro-1H-benzo[d]imidazole-5-carboxamide) was custom synthesized by ChemBridge.

### Animals

All mice were housed at NC State University and all experiments were IACUC approved (protocols 22–167-B, 19 –047-B, and 16–038-B). Mice were given food and water *ad libitum* and kept on a 12-hour light dark cycle (6am –6pm EST).

All inbred mouse lines were bred in-house from mice obtained from The Jackson Laboratory or the NIH. The following lines were purchased from the Jackson Laboratory: C57Bl6/J (Strain # 000664), B6.Cg-*Tac1*^*tm1Bbm*^/J (Substance P (SP) KO) (Strain # 004103)^21^, *Sst*^*tm2.1(cre)Zjh*^/J (SST-cre) (Strain # 013044)^49^, and B6.Cg-*Gt(ROSA)26Sor*^*tm9(CAG0tdTomato)Hze*^/J (Ai9) (Strain # 007909)^50^. BNP KO mice were a generous gift from Dr. Mark Hoon (NIH)^22^. Crl:CD1(ICR) (CD-1) mice were ordered from Charles River laboratories (Strain # 022). SST-cre::Ai9 mice were generated by crossing the SST-cre and Ai9 lines.

### Itch Behavior

Histamine injection: All mice received intradermal injections of 100 μg/20 μL histamine in sterile 1X phosphate buffered saline (1X PBS). This concentration and volume were used for all intradermal injections. Immethridine and 2-Pyridylethylamine were intradermally injected with the same 100 μg/20 μL concentration. Mice were injected in either Dorsal Nape^11^ or Cheek^12^. L-733,060 was dissolved in sterile 1X PBS and 50 μL of a 1mg/kg solution was administered intravenously 30 minutes prior to histamine injection^31,32^. JS-11 was dissolved in DMSO, and the injection solution consisted of 10% Tween-80, 20% DMSO, and 70% Normal saline. A total of 16.3 μg/50 μL of JS-11 was administered intraperitoneally 30 minutes prior to histamine injection^33^.

The same mice were used for both the Cheek and Nape tests. For drug administration injections, mice were randomly assigned to receive Vehicle, L733–060, orJS-11. Mice were then randomized to the injection site. After a 10-day washout period, mice were treated with vehicle were then treated with JS-11 or L-733,060 and mice treated with JS-11 or L-733,060 were treated with vehicle.

### Immunohistochemistry

For Tuj antibody staining, DRG and TG were isolated from SST-cre::Ai9 mice, fixed in 4% PFA for 24 hours at 4°C, cryoprotected using a 30% sucrose solution incubation (~ 72 hrs at 4°C), and then frozen in OTC media. Sections were cut at 14 μm and placed in 4% PFA for 15 minutes at room temperature. Slides were then blocked with solution of 5% BSA with 0.1% Triton-X100. Rabbit Anti-mouse Tuj (Abcam, Catalog # ab18207) (1:500 in 5% blocking solution) was applied to slides, which were then incubated overnight at 4°C. After 16–24 hrs, the slides were washed in ice cold 1X PBS trice and then incubated for 45 minutes at room temperature with Alexa Fluor 488 conjugated goat anti-rabbit IgG (Thermofisher, catalog # A-11008). Samples were images on a Leica DM500B epifluorescent microscope.

### Skin Innervation Tracing

Mice were briefly anesthetized with isoflurane prior to injection of wheat germ agglutinin (WGA) that was conjugated to Alexa Fluor 350 (Invitrogen, Catalog # W11263) (WGA-350) to determine changes in the population sizes of skin-innervating DRG and TG neurons^52^. WGA-350 was injected intradermally into both cheeks and the dorsal nape. Both TG and C3-T2 DRG were isolated 24 hours after injection^52^. TG and DRG were then placed in OCT media and quickly frozen on dry ice and were stored at −80°C for up to 3 months. Cheek injections were bilateral to obtain the greatest potential number of TG sensory neurons that innervate the skin.

#### Fluorescent In Situ Hybridization

In situ hybridization was carried out using the RNAscope Multiplex Fluorescent Reagent Kit v2 (ACD, Catalog # 323100). DRG and TG were isolated from 8-week-old mice 24 hours after receiving 50 μL of a 1mg/mL solution of Alexa Flour-conjugated Wheat Germ Agglutinin (WGA-350) and frozen in OTC media at −80°C within 30 minutes. These sections were cut at thickness of 10 μm and placed onto Superfrost Gold Plus Slides (Fisher, cat. # 15–188-48). After sectioning, sections were air dried at −20°C for 2 hours. After this, slides were placed in 4% paraformaldehyde (4% PFA) made with DEPC water (Thermofisher, Catalog # 750023) at 4°C for 15 minutes. Slides were then washed twice in 1X PBS made with DEPC water. Following this wash step, slides were dehydrated at room temperature in 50% ethanol for 5 minutes, 70% ethanol for 5 minutes, and two washes in 100% ethanol for 5 minutes. The 50% ethanol and 70% ethanol solutions were made with DEPC water. The slides were then kept at −20°C in 100% ethanol overnight. Ethanol was purchased from Millipore Sigma (Catalog # 459836–2L).

The following day, the slides were removed from 100% ethanol and allowed to air-dry at room temperature for 5 minutes. A hydrophobic barrier was drawn around the samples using a hydrophobic barrier pen (ACD, Catalog # 310018). The hydrophobic barrier was allowed to dry for 5 minutes. After drying, roughly 5 drops of RNAscope hydrogen peroxide was added to fill the area within the hydrophobic barrier on the slide and incubated for 10 minutes at room temperature. The slides were then washed in DEPC treated water. After this wash, roughly 5 drops of RNAscope Protease III was added to fill the area within the hydrophobic barrier and incubated for 30 minutes at room temperature. The slides were then washed twice in 1X PBS made with DEPC water. Sections were hybridized and on the same day; therefore, sodium citrate was not used.

For hybridization, probes the following probes were ordered from ACD: Human Tac1 (Substance P) (Catalog# 310711-C3), human SST (Catalog # 310591-C2), human HRH1 (Catalog # 416501), human HRH3 (Catalog # 402191), mouse Tac1 (Catalog # 410351-C2), mouse SST (Catalog # 404631-C3), mouse Hrh1 (Catalog #491141), and mouse Hrh3 (Catalog # 428481). All DRG and TG slides were stained with the corresponding species-specific probes (human or mouse). All slides were stained with SST and Tac1, and either Hrh1 or Hrh3. To hybridize the probes, the hydrophobic barrier area was filled with ~ 6 drops of the appropriate probe mixture and then incubated for 2 hours at 40°C in a HybEZ oven. After this incubation, slides were washed in 1X wash buffer twice for two minutes (each wash). For the first two amplification steps, ~ 6 drops of RNAscope Multiplex FL v2 Amp1 or Amp2 was added to the area within the hydrophobic barrier and incubated for 30 minutes at 40°C. For the third hybridization step, RNAscope Multiplex v2 Amp3 was added to the area within the hydrophobic barrier and incubated for 15 minutes at 40°C. After each hybridization step slides were washed twice in 1X was buffer for 2 minutes at room temperature.

To develop the HRP signal the following steps were performed: roughly 6 drops of RNAscope Multiplex FL v2 HRP-C1, HRP-C2, or HRP-C3 were added to the area within the hydrophobic barrier on the slide and incubated for 15 minutes at 40°C. Slides were then washed twice for 2 minutes in 1X wash buffer at room temperature. To the area in the hydrophobic barrier 200 μL of Opal 520 (channel 1) (Akoya Biosciences, Catalog # FP1487001KT), Opal 570 (channel 2) (Akoya Biosciences, Catalog # FP1488001KT), or Opal 690 (channel 3) (Akoya Biosciences, Catalog # FP1497001KT) was added and the slides were incubated at 40°C for 30 minutes. All dyes were diluted 1:1000. After incubation, slides were washed twice in 1X wash buffer for 2 minutes at room temperature. Finally, roughly 6 drops of RNAscope Multiplex FL v2 HRP blocker was added to the area within the hydrophobic barrier and incubated at 15 minutes at 40°C. After this incubation, the slides were washed twice in 1X wash buffer for 2 minutes at room temperature.

### Cell Counting and Quantification:

Images were collected on a Leica DMB500 epifluorescence microscope. Cells were counted based on appearance since DRG and TG neurons have distinctly round to ovoid shapes with a large round central gap where the nucleus is located. Skin innervating neurons were normalized to the total number of WGA-350 neurons. In Figs. 5, 8, and 9 cell populations were normalized to the total number of cells counted. In Fig. 2, the percentage of SST-expressing neurons was normalized to the total number of TUJ positive neurons counted.

### Statistical Analysis:

Data was analyzed in Graphpad Prism. Paired 2-tailed t-tests were performed to compare histamine-induced scratching in the cheek versus dorsal nape. Region-based data was analyzed with a 2-way ANOVA with a *post hoc* Holm – Šídák test for multiple comparisons. Other data was compared with a 1-way ANOVA with a *post hoc* Holm – Šídák test for multiple comparisons. Literature data was compared using an unpaired 2-tailed Mann-Whitney U-test. For all tests the initial significance value was set to α = 0.05.

## References

[1] Goswami SC (2014) Molecular signatures of mouse TRPV1-lineage neurons revealed by RNA-Seq transcriptome analysis. J Pain 15:1338–1359 25281809 10.1016/j.jpain.2014.09.010 PMC5469214

[2] Unbiased classification of sensory neuron types by large-scale single-cell RNA sequencing | Nature Neuroscience. https://www.nature.com/articles/nn.3881 10.1038/nn.3881 25420068

[3] Goswami SC (2014) Itch-associated peptides: RNA-Seq and bioinformatic analysis of natriuretic precursor peptide B and gastrin releasing peptide in dorsal root and trigeminal ganglia, and the spinal cord. Mol Pain 10:44 25123163 10.1186/1744-8069-10-44 PMC4132360

[4] Flegel C (2015) RNA-Seq Analysis of Human Trigeminal and Dorsal Root Ganglia with a Focus on Chemoreceptors. PLoS ONE 10:e0128951 26070209 10.1371/journal.pone.0128951 PMC4466559

[5] Ray P (2018) Comparative transcriptome profiling of the human and mouse dorsal root ganglia: an RNA-seq-based resource for pain and sensory neuroscience research. Pain 159:1325–1345 29561359 10.1097/j.pain.0000000000001217 PMC6008200

[6] Manteniotis S (2013) Comprehensive RNA-Seq Expression Analysis of Sensory Ganglia with a Focus on Ion Channels and GPCRs in Trigeminal Ganglia. PLoS ONE 8:e79523 24260241 10.1371/journal.pone.0079523 PMC3832644

[7] Nguyen MQ, Wu Y, Bonilla LS, von Buchholtz LJ, Ryba N (2017) J. P. Diversity amongst trigeminal neurons revealed by high throughput single cell sequencing. PLoS ONE 12:e0185543 28957441 10.1371/journal.pone.0185543 PMC5619795

[8] Rukwied R, Zeck S, Schmelz M, McGlone F (2002) Sensitivity of human scalp skin to pruritic stimuli investigated by intradermal microdialysis in vivo. J Am Acad Dermatol 47:245–250 12140471 10.1067/mjd.2002.120461

[9] Truini A (2011) Topographical distribution of warmth, burning and itch sensations in healthy humans. Neurosci Lett 494:165–168 21396428 10.1016/j.neulet.2011.03.004

[10] Bin Saif GA (2012) Aberrant C nerve fibre function of the healthy scalp. Br J Dermatol 167:485–489 22633050 10.1111/j.1365-2133.2012.11070.x

[11] Kuraishi Y, Nagasawa T, Hayashi K, Satoh M (1995) Scratching behavior induced by pruritogenic but not algesiogenic agents in mice. Eur J Pharmacol 275:229–233 7539379 10.1016/0014-2999(94)00780-b

[12] Shimada SG, LaMotte RH (2008) Behavioral differentiation between itch and pain in mouse. Pain 139:681–687 18789837 10.1016/j.pain.2008.08.002 PMC2723192

[13] Tsujii K, Andoh T, Lee J-B, Kuraishi Y (2008) Activation of proteinase-activated receptors induces itch-associated response through histamine-dependent and -independent pathways in mice. J Pharmacol Sci 108:385–388 18987429 10.1254/jphs.08200sc

[14] Liu Q (2009) Sensory neuron-specific GPCR Mrgprs are itch receptors mediating chloroquine-induced pruritus. Cell 139:1353–1365 20004959 10.1016/j.cell.2009.11.034 PMC2989405

[15] Akiyama T, Carstens MI, Carstens E (2010) Facial Injections of Pruritogens and Algogens Excite Partly Overlapping Populations of Primary and Second-Order Trigeminal Neurons in Mice. J Neurophysiol 104:2442 20739601 10.1152/jn.00563.2010 PMC3350035

[16] Akiyama T, Carstens MI, Carstens E (2010) Enhanced scratching evoked by PAR-2 agonist and 5-HT but not histamine in a mouse model of chronic dry skin itch. Pain 151:378 20709455 10.1016/j.pain.2010.07.024 PMC2955821

[17] Akiyama T, Carstens MI, Carstens E (2010) Differential itch- and pain-related behavioral responses and μ-opoid modulation in mice. Acta Derm Venereol 90:575–581 21057739 10.2340/00015555-0962

[18] A subpopulation of nociceptors specifically linked to itch |. Nat Neurosci. https://www.nature.com/articles/nn.3289 10.1038/nn.3289 PMC3557753 23263443

[19] Kardon AP (2014) Dynorphin Acts as a Neuromodulator to Inhibit Itch in the Dorsal Horn of the Spinal Cord. Neuron 82:573–586 24726382 10.1016/j.neuron.2014.02.046 PMC4022838

[20] Huang J (2018) Circuit dissection of the role of somatostatin in itch and pain. Nat Neurosci 21:707–716 29556030 10.1038/s41593-018-0119-z PMC5923877

[21] Cao YQ (1998) Primary afferent tachykinins are required to experience moderate to intense pain. Nature 392:390–394 9537322 10.1038/32897

[22] Mishra SK, Hoon MA (2013) The Cells and Circuitry for Itch Responses in Mice. Science 340:968–971 23704570 10.1126/science.1233765 PMC3670709

[23] A gastrin-releasing peptide receptor mediates the itch sensation in the spinal cord | Nature. https://www.nature.com/articles/nature06029 10.1038/nature06029 17653196

[24] Suga S (1992) Receptor selectivity of natriuretic peptide family, atrial natriuretic peptide, brain natriuretic peptide, and C-type natriuretic peptide. Endocrinology 130:229–239 1309330 10.1210/endo.130.1.1309330

[25] Regoli D, D’Orlrans-Juste P, Rouissi N, Rhaleb N. E. Vasoactive peptides and characterization of their receptors10.1016/0167-0115(93)90359-g 8394593

[26] Wheeler JJ, Allen-Moyer KN, Mishra S (2020) K. A systematic review of animal models and sex as a variable in itch research. Itch 5:e40

[27] Trigeminal activation patterns evoked by chemical stimulation of the dura mater in rats - PMC. https://pmc.ncbi.nlm.nih.gov/articles/PMC7429748/10.1186/s10194-020-01169-4 PMC7429748 32799798

[28] Bogusch G (1987) Innervation of the dermatomes in the neck of the mouse. Acta Anat (Basel) 129:275–278 3630614 10.1159/000146415

[29] Solinski HJ (2019) Nppb Neurons Are Sensors of Mast Cell-Induced Itch. Cell Rep 26:3561–3573e4 30917312 10.1016/j.celrep.2019.02.089 PMC6490177

[30] Rupniak NMJ, Carlson E, Boyce S, Webb JK, Hill RG (1996) Enantioselective inhibition of the formalin paw late phase by the NK1 receptor antagonist L-733,060 in gerbils. Pain 67:189–195 8895247 10.1016/0304-3959(96)03109-0

[31] Varty GB (2002) The gerbil elevated plus-maze II: anxiolytic-like effects of selective neurokinin NK1 receptor antagonists. Neuropsychopharmacol Off Publ Am Coll Neuropsychopharmacol 27:371–379 10.1016/S0893-133X(02)00313-5 12225694

[32] Solinski HJ (2019) Inhibition of natriuretic peptide receptor 1 reduces itch in mice. Sci Transl Med 11:eaav5464 31292265 10.1126/scitranslmed.aav5464 PMC7218920

[33] Durant GJ, Ganellin CR, Parsons ME (1975) Chemical differentiation of histamine H1- and H2-receptor agonists. J Med Chem 18:905–909 240025 10.1021/jm00243a009

[34] Kitbunnadaj R (2004) Identification of 4-(1H-imidazol-4(5)-ylmethyl)pyridine (immethridine) as a novel, potent, and highly selective histamine H(3) receptor agonist. J Med Chem 47:2414–2417 15115383 10.1021/jm049932u

[35] Aldinger KA, Sokoloff G, Rosenberg DM, Palmer AA, Millen KJ (2009) Genetic Variation and Population Substructure in Outbred CD-1 Mice: Implications for Genome-Wide Association Studies. PLoS ONE 4:e4729 19266100 10.1371/journal.pone.0004729 PMC2649211

[36] McMahon SB, Koltzenburg M (1992) Itching for an explanation. Trends Neurosci 15:497–501 1282750 10.1016/0166-2236(92)90102-e

[37] Schmelz M, Schmidt R, Bickel A, Handwerker HO, Torebjörk HE (1997) Specific C-receptors for itch in human skin. J Neurosci Off J Soc Neurosci 17:8003–8008 10.1523/JNEUROSCI.17-20-08003.1997 PMC6793906 9315918

[38] Fatima M (2019) Spinal somatostatin-positive interneurons transmit chemical itch. Pain 160:1166–1174 30913166 10.1097/j.pain.0000000000001499

[39] Zhang X, Tong Y-G, Bao L, Hökfelt T (1999) The neuropeptide Y Y1 receptor is a somatic receptor on dorsal root ganglion neurons and a postsynaptic receptor on somatostatin dorsal horn neurons. Eur J Neurosci 11:2211–2225 10383610 10.1046/j.1460-9568.1999.00638.x

[40] Mu D (2017) A central neural circuit for itch sensation. Science 357:695–699 28818946 10.1126/science.aaf4918

[41] Sheahan TD (2023) Identification of a convergent spinal neuron population that encodes itch. 09.29.560205 Preprint at 10.1101/2023.09.29.560205 (2023)

[42] Pain Inhibits GRPR Neurons via GABAergic Signaling in the Spinal Cord | Scientific Reports. https://www.nature.com/articles/s41598-019-52316-0 10.1038/s41598-019-52316-0 PMC6825123 31676846

[43] Sun S (2017) Leaky gate model: intensity-dependent coding of pain and itch in the spinal cord. Neuron 93:840 28231466 10.1016/j.neuron.2017.01.012 PMC5324710

[44] Pagani M (2019) How Gastrin-Releasing Peptide Opens the Spinal Gate for Itch. Neuron 103:102–117e5 31103358 10.1016/j.neuron.2019.04.022 PMC6616317

[45] Rodriguez E (2017) A craniofacial-specific monosynaptic circuit enables heightened affective pain. Nat Neurosci 20:1734 29184209 10.1038/s41593-017-0012-1 PMC5819335

[46] Ding YQ, Takada M, Shigemoto R, Mizuno N (1995) Trigeminoparabrachial projection neurons showing substance P receptor-like immunoreactivity in the rat. Neurosci Res 23:415–418 8602281 10.1016/0168-0102(95)00961-R

[47] Carter ME, Soden ME, Zweifel LS, Palmiter R (2013) D. Genetic identification of a neural circuit that suppresses appetite. Nature 503:111–114 24121436 10.1038/nature12596 PMC3878302

[48] Taniguchi H (2011) A resource of Cre driver lines for genetic targeting of GABAergic neurons in cerebral cortex. Neuron 71:995–1013 21943598 10.1016/j.neuron.2011.07.026 PMC3779648

[49] Madisen L (2010) A robust and high-throughput Cre reporting and characterization system for the whole mouse brain. Nat Neurosci 13:133–140 20023653 10.1038/nn.2467 PMC2840225

[50] Chen Y (2020) The prolactin receptor long isoform regulates nociceptor sensitization and opioid-induced hyperalgesia selectively in females. Sci Transl Med 12:eaay7550 32024801 10.1126/scitranslmed.aay7550 PMC7523341

[51] Johnson ND, Duysen EG, Lockridge O (2009) Intrathecal delivery of fluorescent labeled butyrylcholinesterase to the brains of butyrylcholinesterase knock-out mice: Visualization and quantification of enzyme distribution in the brain. Neurotoxicology 30. 10.1016/j.neuro.2009.03.001 PMC3864044 19442823

[52] von Buchholtz LJ, Lam RM, Emrick JJ, Chesler AT, Ryba N (2020) J. P. Assigning transcriptomic class in the trigeminal ganglion using multiplex in situ hybridization and machine learning. Pain 161:2212 32379225 10.1097/j.pain.0000000000001911 PMC7606614

